# Silica Nanochannel Array Film Supported by *ß*-Cyclodextrin-Functionalized Graphene Modified Gold Film Electrode for Sensitive and Direct Electroanalysis of Acetaminophen

**DOI:** 10.3389/fchem.2021.812086

**Published:** 2022-01-13

**Authors:** Huaxu Zhou, Yao Ding, Ruobing Su, Dongming Lu, Hongliang Tang, Fengna Xi

**Affiliations:** ^1^ Department of Chemistry, Key Laboratory of Surface and Interface Science of Polymer Materials of Zhejiang Province, Zhejiang Sci-Tech University, Hangzhou, China; ^2^ Guangxi University of Chinese Medicine, Nanning, China; ^3^ The First Affiliated Hospital of Guangxi University of Chinese Medicine, Nanning, China; ^4^ Affiliated Fangchenggang Hospital, Guangxi University of Chinese Medicine, Fangchenggang, China

**Keywords:** electrochemical detection, silica nanochannel array film, conductive adhesion layer, *ß*-cyclodextrin modified reduced graphene oxide, acetaminophen

## Abstract

Convenient and sensitive detection of active analytes in complex matrix is crucial in biological, medical, and environmental analysis. Silica nanochannel array film (SNF) equipped electrochemical sensors have shown excellent anti-fouling performance in direct analysis of complex samples. In this work, we demonstrated an electrochemical sensor with anti-fouling performance for highly sensitive detection of acetaminophen (APAP) based on SNF supported by *ß*-cyclodextrin-graphene (CDG) nanocomposite modified Au film electrode (AuF). Because of their rich surface hydroxyls and 2D lamellar structure, CDG on AuF can serve as the nanoadhesive for compact binding SNF, which can be grown by electrochemical assisted self-assembly method in a few seconds. Attributable to the electrocatalytic property of graphene and the synergistic enrichment from both CD and SNF nanochannels towards analyte, the SNF/CDG/AuF sensor demonstrates sensitive detection of acetaminophen ranged from 0.2 to 50 μM with an ultralow limit-of-detection of 14 nM. Taking advantage of the anti-fouling ability of SNF, the sensor is able to realize accurate and convenient analysis of APAP in commercially available paracetamol tablets.

## Introduction

Electrochemical sensors have attracted much attention owing to their high sensitivity, simple operation, and no need of expensive instruments (Bollella et al., 2017; Cesewski and Johnson, 2020; Bagheri et al., 2021; Mehmandoust et al., 2021; Min et al., 2021; Karimi-Maleh et al., 2022). However, direct electrochemical analysis of complex samples is challenging because of anti-fouling or anti-interference from the complicated matrix (Yan et al., 2016; Lu et al., 2018). Recently, silica nanochannel array film (SNF) has been proven to be an effective anti-fouling and anti-inference layer to functionalize the supporting electrodes. SNF is an ultrathin nanofilm with highly uniform and high density nanochannels perpendicular to the supporting electrode (Poltorak et al., 2013; Ma et al., 2020; Ding et al., 2021; Ullah et al., 2021; Yan et al., 2021). The unique structure confers SNF with excellent molecular screening ability based on size and charge of molecules. On the one hand, most biological macromolecules or other large substances in complex samples have difficulties in entering the ultrasmall nanochannels of SNF, therefore providing outstanding anti-fouling ability. On the other hand, SNF is rich in Si-OH groups (p*K*_a_ ∼ 2), leading to negatively charged surface in conventional solution medium (Zhou et al., 2015; Guo et al., 2016; Liu et al., 2016; Yan and Su, 2016; Ding et al., 2020). Therefore, SNF modified electrode has great potential in direct and sensitive electroanalysis of complex samples.

Until now, three sensing strategies have been commonly used to fabricate SNF-based sensors. Generally, SNF can be conveniently synthesized using surfactants (e.g., cetyltrimethylammonium bromide, CTAB) as the template through electrochemical assisted self-assembly (EASA) method or Stöber solution growth method (Yan et al., 2016; Liu et al., 2017; Liang et al., 2021). Thus, the first sensing strategy is to retain the surfactant micelle (SM) in the nanochannels and use them as extracting agents (Yan et al., 2016). However, SM-based SNF sensor can only detect neutral or organic small molecules with electrical activity. The second sensing strategy is to remove SM and functionalize the open nanochannels. The derivatization of nanochannels is mainly realized through the introduction of organic functional groups or nanomaterials. Generally, SNF containing organic reaction groups (e.g., -NH_2_ or -SH) can be synthesized through co-polycondensation of silanes precursors containing functional groups (Yan et al., 2016; Nasir et al., 2019; Nasir et al., 2020; Zhou et al., 2020). Subsequent reactions with -NH_2_ or -SH, organic molecules (e.g., ferrocene, tetrazine, hydroquinone, etc.) or biological ligands (e.g., aptamer) can be further introduced on nanochannels. The reported nanomaterials to modified SNF nanochannels includes metal nanoparticles, nanowires, graphene quantum dots, conductive polymers, etc. For example, Chen group electrochemically deposited gold nanoparticles (AuNPs) with a particle size of 1.6 nm in NH_2_ modified SNF (NH_2_-SNF) (Wu et al., 2015). Su group also synthesized AuNPs in NH_2_-SNF based on electrostatic adsorption of AuCl_4_^−^ into nanochannels followed by chemical reduction with NaBH_4_ (Ding et al., 2014). Our group confined graphene quantum dot (GQD) into SNF nanochannels to achieve ultrasensitive detection of heavy metal ions (e.g., Hg^2+^) (Lu et al., 2018). In addition, conductive polymers such as polypyrrole, polyaniline, and polythiophene are also reported to modify SNF nanochannels (Yan et al., 2016). However, accurate control of the size of nanomaterials is crucial so that they do not affect diffusion.

Different from the above two methods based on the introduction of functional ligands inside the nanochannels, the third strategy is to introduce functionality on the supporting electrode substrate. Due to poor adhesion, SNF can not be stably bonded on common carbon (e.g., glassy carbon electrode-GCE or carbon fiber electrode) or metal (e.g., Au or Pt) electrodes without molecular glue (e.g., 3-aminopropyltriethoxysilane-APTES or 3-mercaptopropyltriethoxysilane). Therefore, most SNF sensors use ITO as the supporting electrode because it is possible to achieve stable adhesion through Si-O-In or Si-O-Sn bonds (Wu et al., 2015; Yan et al., 2016; Lu et al., 2018). However, ITO often has high overpotential for many redox organic molecules. To expand the supporting substrate, our group applied chemically or electrochemically reduced graphene oxide (rGO) as conductive layer to fabricated SNF modified GCE, which led to improved detection sensitivity and lower oxidation potential towards several analytes (Cheng et al., 2018). We also used electro-activated GCE as the supporting electrode to grow SNF. Due to the introduction of functional oxygen-containing groups into electro-activated GCE, the fabricated sensor exhibited high electrocatalytic performance (Xuan et al., 2021). Compared with methods based on modifying nanochannels to introduce functionality, these strategies using functionally supporting electrode substrates are simpler and more convenient. On the one hand, the functionalization of the supporting electrode will not affect the size and structure of SNF nanochannels, so it will not cause plugging or reduce substance diffusion. On the other hand, the introduction of functional materials does not need to be restricted by the ultrasmall size of nanochannels, which is beneficial to the integration of a wide range of functional materials. However, the existing work is limited and focuses on the improvement of electrocatalytic performance including reducing the detection potential or improving the potential resolution. Further introducing a wide range of functionalized elements on the supporting electrode substrate is highly desirable to expand the application of SNF sensors.

**FIGURE 1:**
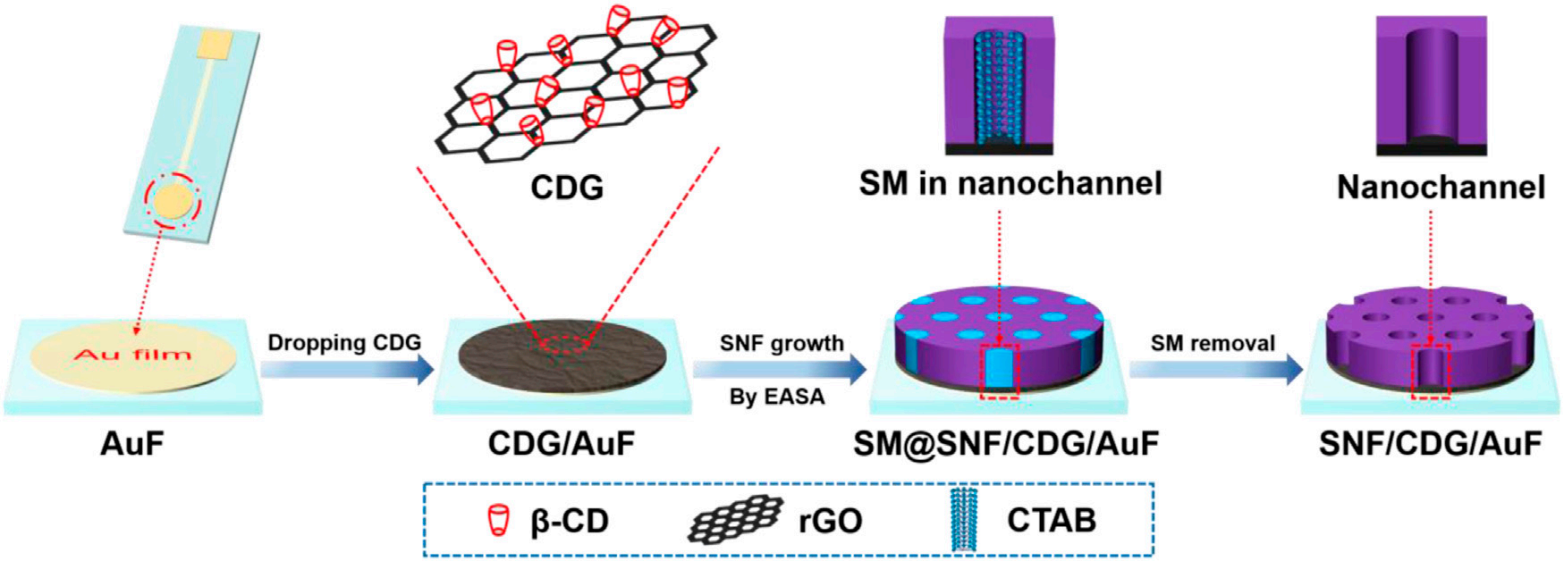
Illustration of facile preparation of SNF/CDG/AuF by EASA method using CDG as nanoadhesive.

In this work, we demonstrate the integration of SNF with *ß*-cyclodextrin-graphene (CDG) nanocomposite modified Au film electrode (AuF) for sensitive and direct electroanalysis of acetaminophen, an antipyretic analgesic. As an oligosaccharide comprises seven glucose units, *ß*-cyclodextrin with unique hydrophobic inner cavity and hydrophilic exterior has high recognition and enrichment capabilities based on host-guest interaction or supramolecular assembly (Guo et al., 2010; Guo et al., 2011; Chen et al., 2013; Mirzaei et al., 2021). As shown in Figure 1, SNF is grown using EASA method after dropping CDG on AuF. On the one hand, -OH groups of CD provide hydrogen bond or covalent bonding interaction with SNF, leading to high adhesion of SNF. On the other hand, enrichment from CD by forming guest-host complex and SNF by electrostatic adsorption also facilitates sensitive detection of acetaminophen. Combined with anti-fouling ability of SNF, the developed sensor realizes sensitive and direct electroanalysis of acetaminophen in paracetamol tablets.

## Materials and Methods

### Chemicals and Materials

Au film electrode (AuF) was obtained from Beijing Reizhi Hanxing Technology Co., Ltd (China). Graphene oxide (GO) solution (10 mg/g) was purchased from GaoxiTech Co., Ltd (China). Tetraethyl orthosilicate (TEOS, with a purity of 98%), Na_2_HPO_4_, NaH_2_PO_4_, cetyltrimethylammonium bromide (CTAB, with a purity of 99%), acetaminophen (APAP), p-aminophenol (4-AP), potassium hydrogen phthalate (KHP), potassium ferricyanide (K_3_Fe(CN)_6_), *ß*-cyclodextrin (*β*-CD, with a purity of 98%), and hydrazine hydrate (50%) were purchased from Aladdin Biochemical Technology Co., Ltd (China). Ruthenium hexaammoniate trichloride (Ru(NH_3_)_6_Cl_3_) and ammonia (25%) were purchased from sigma-Aldrich (United States). APAP paracetamol tablets were purchased from Tianjin Jiansheng Pharmaceutical Co., Ltd (China). All chemicals and reagents of analytical grade were used as received without further purification. Ultrapure water used in the experiment was prepared using Mill-Q system (Millipore, United States).

### Measurements and Instrumentations

X-ray photoelectron spectroscopy (XPS) measurements were performed using Mg K*α* as excitation (PHI5300, PE Ltd, United States). UV-vis spectra were recorded on a UV-2450 spectrophotometer (Shimadzu Corporation, Japan). Electrochemical experiments including cyclic voltammetry (CV), differential pulse voltammetry (DPV), and electrochemical impedance spectroscopy (EIS) were carried out on an AutoLab station (PGSTAT302N, Metrohm, Switzerland) using three electrode system. Briefly, bare or modified AuF electrode was applied as the working electrode. Platinum wire and Ag/AgCl electrode (saturated KCl solution) were used as the auxiliary and reference electrode, respectively. The scan rate in CV was 100 mV/s. The step potential for DPV measurement was 5 mV with a pulse amplitude of 25 mV and pulse time of 0.05 s (interval 0.2 s). EIS investigation was performed in 0.1 M KCl solution containing 2.5 mM K_3_Fe(CN)_6_ and 2.5 mM K_4_Fe(CN)_6_. The frequencies range from 10^4^ to 10^–1^ Hz. Transmission electron microscopy (TEM) images were obtained from JEM-2100 (JEOL, Japan) at operating voltage of 200 kV. SNF was mechanically scrapped from the surface of AuF electrode and dispersed in ethanol by sonication. Then SNF dispersion was dropped onto the copper grid. Fourier transform infrared spectroscopy (FTIR) were obtained using Vertex 70 spectrometer (Bruker, United States) using KBr tablet method.

### Preparation of CDG

As reported previously (Guo et al., 2010), ammonia (300 μl) was added in the mixture of GO (0.25 mg/ml, 20 ml) and *ß*-CD (0.2 mg/ml, 20 ml). After adding 20 μL hydrazine hydrate solution, the solution was reacted at 60°C under stirring for 3.5 h. The obtained black solution was centrifuged at 10,000 rpm for 30 min. The precipitate was washed thoroughly and then re-dispersed in water to obtain CDG solution. For comparison, rGO was prepared using the same procedure without adding *ß*-CD.

### Preparation of SNF/CDG/AuF

Before use, bare AuF was firstly electrochemical polished using cyclic voltammetry scanning (1.6 V ∼ −0.2 V) in H_2_SO_4_ (0.1 M). After dropping CDG dispersion (10 μL, 0.1 mg/ml) on AuF surface, the obtained CDG/AuF was dried at 60°C. To grow SNF, silica precursor solution containing CTAB (1.585 g), TEOS (3 mL), and ethanol (20 ml) was prepared in sodium nitrate solution (20 ml, 0.1 M, pH = 2.6). The obtained solution was pre-hydrolysed for 2.5 h with stirring to prepare the precursor solution. When CDG/AuF was immersed in the precursor solution, a negative potential (−2.2 V) was applied for 5 s. Then, the obtained electrode was thoroughly washed with ultrapure water and aged at 80°C overnight. SM in nanochannels was removed using the mixture of HCl (0.1 M) and ethanol (*V*:*V* = 1:1) and the resulting electrode was termed as SNF/CDG/AuF. For comparison, rGO was applied as the adhesion layer and the same procedure was applied to prepare SNF/rGO/AuF as the control.

### Electrochemical Detection of APAP

PBS (0.1 M, pH 3.0) was used as supporting electrolyte for detection. DPV curves of APAP at SNF/CDG/AuF were measured after adding different concentrations of APAP. In order to evaluate the detection selectivity, different concentrations of ions, biologically related interfereces and p-aminophenol (4-AP) were added in APAP solution (10 μM) and the corresponding DPV curve was measured. For real sample analysis, APAP paracetamol tablets were ground into powder. The obtained powder (0.4 g) was added to ethanol (10 ml). The resulting suspension was centrifugated for 30 min at 3,000 rpm. The obtained supernatant was diluted using the supporting PBS solution (*V*:*V* = 1:1,000). The concentration of APAP is also detected using high performance liquid chromatography (HPLC, Agilent 1,260 Series) equipped with ZORBAX SB-C18 column (5 μm, 4.6 × 250 mm) and UV detector at 245 nm (mobile phase: 0.05 M ammonium acetate/methanol, V: V = 85:15; flowing rate: 1 mL/min, column temperature: 30°C).

## Results and Discussion

### Integration of Stable SNF on AuF Using CDG as Nanoadhesive

Au film electrode (AuF) has good electrochemical performance and possesses unique advantages of simple preparation, easy integration, and miniaturization. However, SNF exhibits poor adhesion to it. The introduction of a conductive adhesive layer containing functional recognition ability is an effective strategy to improve the stability of SNF and the following detection performance. As we all know, many substances have recognition or other functions, and can be used to disperse reduced graphene oxide (Mirzaei et al., 2021). As shown in Figure 1, *ß*-cyclodextrin-graphene (CDG) nanocomposite is applied as the functional and conductive layer to improve the adhesion of SNF to AuF. On the one hand, CD is well-known owing to its recognition and enrichment capabilities. At the same time, rGO has advantages of high surface area, good conductivity, and outstanding electrocatalytic ability. Thus, CDG shows great potential in sensitive electrochemical detection. At the same time, -OH groups in both CD and rGO facilitate the stable binding of SNF through forming hydrogen bond or covalent Si-O-C bonds. This strategy is significantly different from the existing reports using organosilane (e.g., 3-aminopropyltriethoxysilane-APTES or 3-mercaptopropyltriethoxysilane) as molecular glue. Although the molecular glue can achieve stable adhesion of SNF, its non-conductive properties can not promote electron transfer. As illustrated, SNF can be rapidly grown (5 s) using EASA method after coating CDG on the surface of AuF. The mechanism for the growth is the self-assembly of CTAB surfactant micelle (SM) and the sol-gel reaction of siloxane precursor around SM, which is greatly promoted at local pH gradient formed from reduction of water and protons at low potential (−2.2 V). After the growth, SM is filled in the nanochannel, and the resulting electrode is termed as SM@SNF/CDG/ITO. When the HCl-ethanol mixed solution is used to remove SM, SNF modified AuF with open channels (SNF/CDG/ITO) is obtained.

### Preparation and Characterization of CDG

**FIGURE 2:**
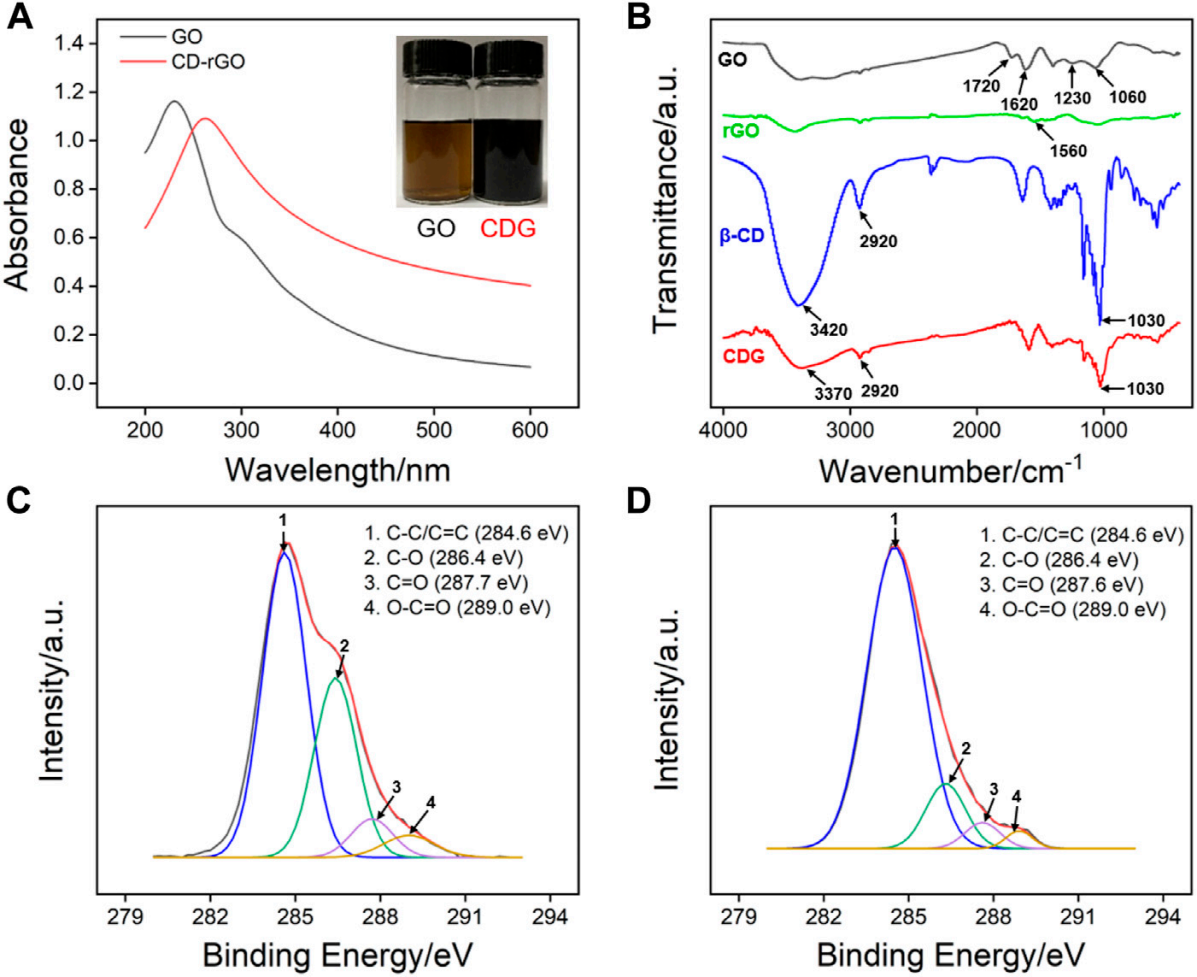
**(A)** UV-vis absorption spectra of GO and CDG. **(B)** FT-IR spectra of GO, rGO, *ß*-CD and CDG. **(C,D)** high-resolution C1s XPS profiles of GO and CDG.

Figure 2A shows the UV-Vis absorption spectra of GO and CDG. Inset in Figure 2A shows that GO is a brown solution, while CDG becomes a black solution. In comparison with GO that has a maximum absorption peak at ∼230 nm, CDG displays a maximum absorption wavelength at ∼263 nm corresponding to *π*-*π* ^*^ transition. In addition, the shoulder peak (290–300 nm) of GO corresponding to *n*-*π* ^*^ electron transition of C=O disappears. The phenomena indicate the successful reduction of GO by hydrazine with a restore of the conjugated structure of graphene. The changes of chemical composition during the preparation of CDG are further confirmed by Fourier transform infrared spectroscopy (FT-IR, Figure 2B) and X-ray photoelectron spectroscopy (XPS, Figures 2C,D). As shown in Figure 2B, the typical signals of C=O stretching (1720 cm^−1^), O-H (3,420 cm^−1^), C=C (1,620 cm^−1^), C-O-C (1,230 cm^−1^), and C-O (1,060 cm^−1^) indicate the presence of high content of oxygen-containing groups. For rGO prepared using the same reduction process without *ß*-CD, the characteristic absorption bands of oxide groups obviously decreased while C=C conjugation (1,560 cm^−1^) increased, suggesting the successful reduction of GO. In case of *ß*-CD, the bands at 2,920 cm^−1^ and 3,420 cm^−1^ are assigned to -CH_2_ asymmetric stretching vibration, and -OH stretching vibration, respectively. The characteristic peaks of *ß*-CD are observed in the spectrum of CDG, indicating that *ß*-CD is attached to rGO. In addition, the band at 3,420 cm^−1^ for the stretching vibrations of–OH in *ß*-CD shifts to 3,370 cm^−1^ in CDG, suggesting the formation of hydrogen bonding in CDG nanocomposite. As shown in high-resolution C1s XPS profiles, there are four types of carbon atoms in GO including C-C/C=C (284.6 eV, sp^2^ C), C-O (286.7 eV), C = O (287.8 eV), and O-C = O (288.7 eV). After GO is reduced, the intensity of the characteristic peak of C-O significantly decreases although CD is bound to rGO by hydrogen bonds. At the same time, the intensity of the characteristic peak of sp (Bollella et al., 2017) C increases, proving the recovery of conjugated structure through reduction and successful synthesis of CDG.

### Characterization of SNF on CDG/AuF

**FIGURE 3:**
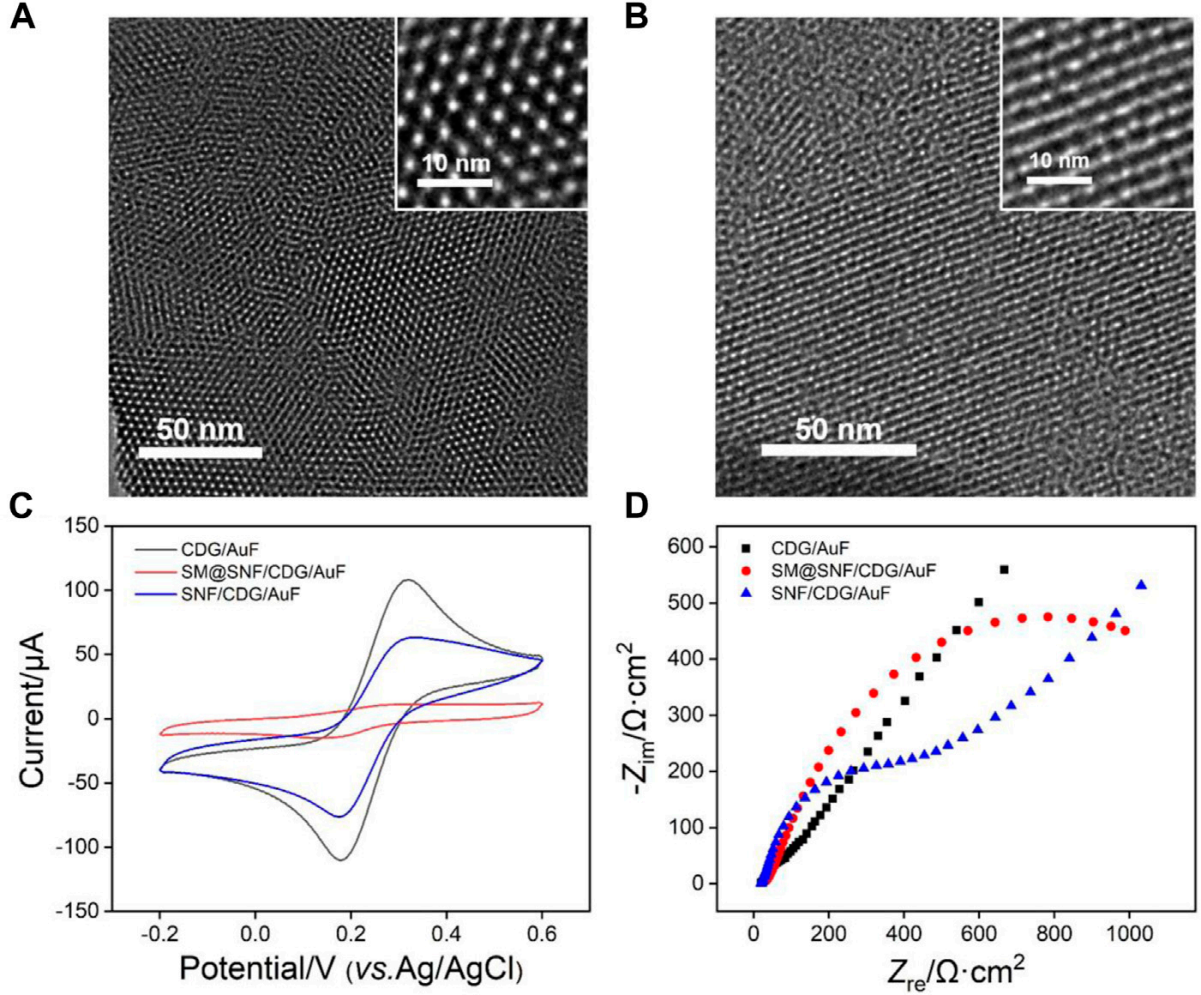
Top-view **(A)** and cross-sectional **(B)** TEM images of SNF. The insets show TEM images at high magnification. **(C)** CV curves obtained at different electrodes in 0.05 M KHP solution containing 0.5 mM Fe(CN)_6_^3+^. The scan rate was 100 mV/s. **(D)** Nyquist plots of different electrode obtained in 0.1 M KCl solution containing 2.5 mM K_3_Fe(CN)_6_ and 2.5 mM K_4_Fe(CN)_6_.

The morphology of SNF was characterized by Transmission electron microscopy (TEM). Figure 3A displays the top-view TEM image of SNF at different magnification. A flat film without crack can be observed in the submicron range. In addition, high density pores are regularly arranged. The inset in TEM image reveals a mesoporous structure with average pore size between 2 and 3 nm. The calculated porosity is ∼44%. As shown in the cross-sectional TEM image (Figure 3B), SNF has nanochannels that are perpendicular to the substrate.

The successful growth of SNF on CDG modified electrode is verified using cyclic voltammetry (CV) measurement of standard electrochemical probe (K_3_Fe(CN)_6_). CV curves obtained on CDG/AuF and SM@CDG/AuF were also measured for comparison. As shown in Figure 3C, SM@SNF/CDG/AuF has almost no redox signals of Fe(CN)_6_^3-^ because SM inside nanochannels blocks the entry of hydrophilic molecules. This proves that SNF grown on CDG/AuF has good integrity. When SM in the nanochannel is removed, the redox probe molecules can enter the nanochannel and undergo electron transfer with the supporting electrode. Compared with CDG/AuF, the redox current on SNF/CDG/AuF slightly reduces. Owing to rich Si-OH groups (p*K*_a_≈2) on SNF, the nanochannels are negatively charged and show significant electrostatic repulsion to the negatively charged probe. This phenomenon was also confirmed by electrochemical impedance spectroscopy (EIS, Figure 3D). As known, the charge transfer resistance (*R*_ct_) of the electrode is related to the diameter of curve semicircle in high frequency region. When SM exists in nanochannels, the SM@SNF/AuF electrode exhibits the largest *R*_ct_ owing to the blocking effect of micelles, which hinders the electron transfer between the electrode and the electrochemical probe. After removing the SM, the *R*_ct_ of SNF/CDG/AuF decreases, but it is larger than that of CDG/AuF. This might be ascribed to the electrostatic repulsion between the negatively charged channels and electrochemical probe.

### Enrichment of APAP by CD and SNF Nanochannels

**FIGURE 4:**
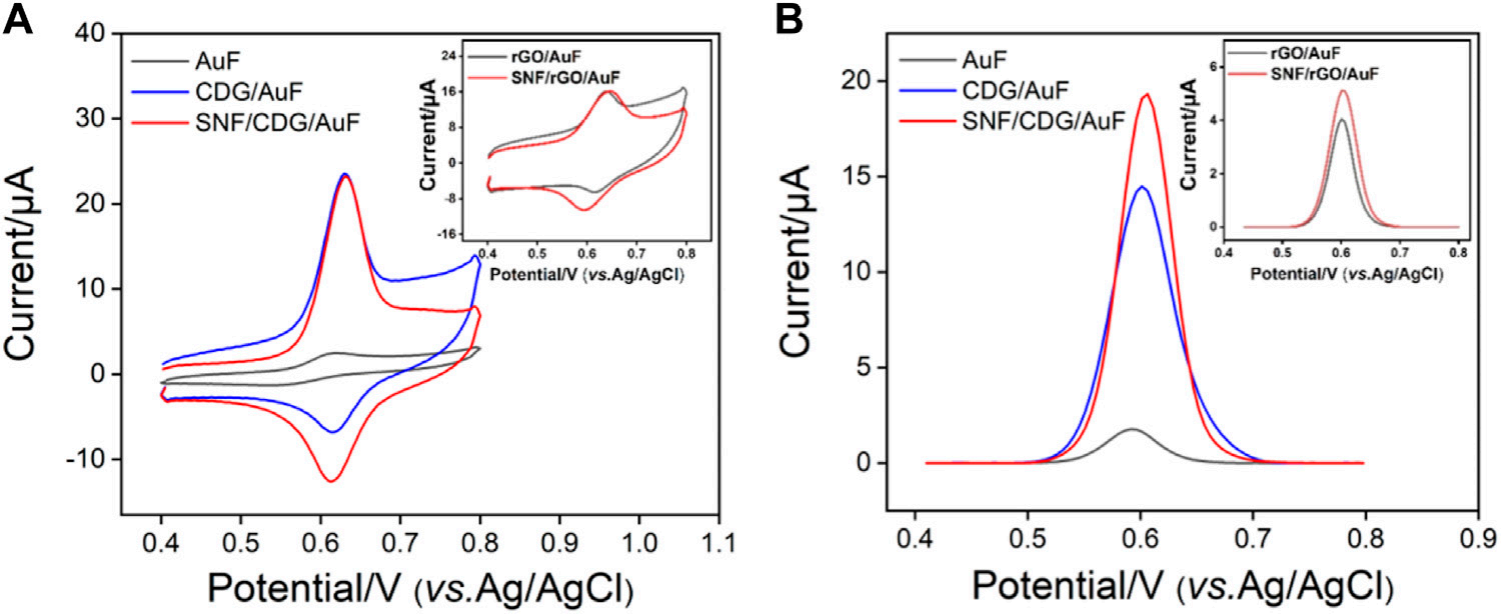
CV **(A)** and DPV **(B)** curves of APAP (20 μM) at different electrodes.

In order to prove the enrichment of APAP by CD and SNF nanochannels, CV or DPV curves of APAP at bare AuF, CDG/AuF, or SNF/CDG/AuF were investigated (Figures 4A,B). For comparison, signals on rGO/AuF or SNF/rGO/AuF without CD were also studied (inset in Figures 4A,B). Compared with bare AuF, both rGO/AuF and CDG/AuF show high redox peaks, resulting from excellent electron conductivity of rGO. Thus, the introduction of rGO on the adhesion layer promotes the electrochemical response of the electrode. At the same time, CDG/AuF displays a higher oxidation peak compared with rGO/AuF, proving the enrichment effect of CD towards APAP resulting from its host-guest complex. It is worth noting that the redox peaks on SNF modified electrode do not decrease although the growth of SNF reduces the effective area of the electrode by 56% as calculated form porosity. Thus, SNF nanochannels also have enrichment towards APAP, which may be due to the electrostatic interaction between the nanochannels and APAP. In other words, SNF/CDG/AuF combines the double enrichment from both CD and SNF, indicating great potential for sensitive detection.

### Optimized Conditions for Electrochemical Detection of APAP

**FIGURE 5:**
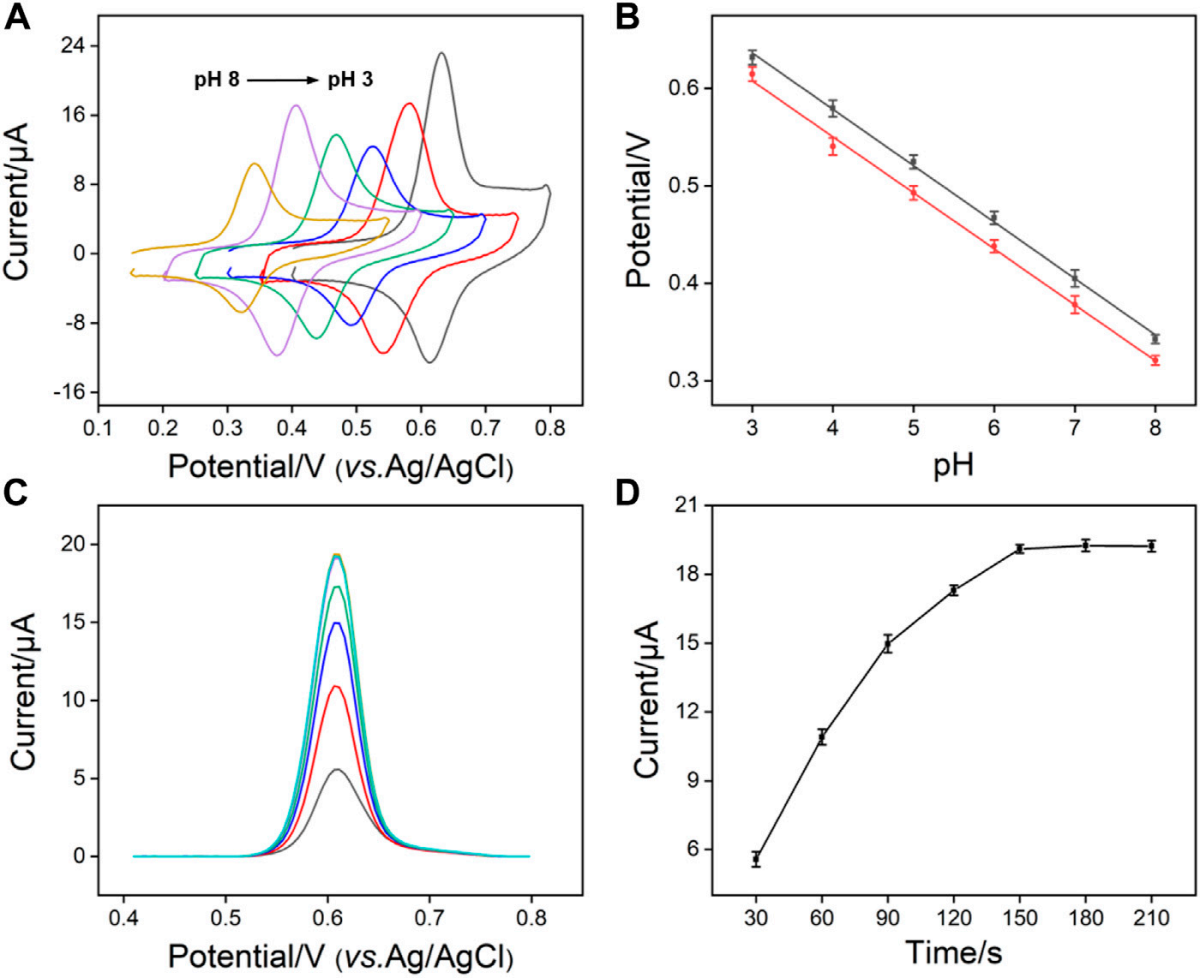
**(A)** CV curves of APAP (20 μM) obtained on SNF/CDG/AuF in 0.1 M PBS at various pH values. **(B)** The plots of anodic and cathodic peak potential with pH values. **(C)** DPV curves and oxidation peak currents **(D)** measured on SNF/CDG/AuF towards APAP (20 μM) in 0.1 M PBS (pH 3.0) at different accumulation time.

In order to obtain the best detection performance, we optimized the pH and enrichment time of the support buffer. Figure 5A depicts the signal of APAP on SNF/CDG/AuF at different pH. As shown, the highest redox peak appears when the pH is 3, resulting from the possible hydrogen bonds between APAP and -OH groups from CD or Si-OH groups from SNF. When pH changes from 3 to 7, the peak current firstly decreases and then increases. With the increase of pH, the negative charge on the inner wall of nanochannel increases, leading to enrichment of APAP by electrostatic attraction. The decrease of peak current at pH eight might be attributed to the slight instability of the film under alkaline conditions. Thus, pH 3 is chosen for further investigation because of the highest enrichment effect. Figure 5B shows the relationship between oxidation or reduction peak currents and pH value. A slope of 58.0 mV/pH is observed, which is close to the slope of Nernst equation of 59.0 mV/pH, indicating that the oxidation-reduction reaction of APAP is a two electron and two proton reactions. Figure 5C gives the DPV curves obtained with the increase of enrichment time. As shown in Figure 5D, the peak current of APAP on SNF/CDG/AuF increases and then reaches the plateau. Therefore, enrichment for 150 s is used as the optimized conditions.

**FIGURE 6:**
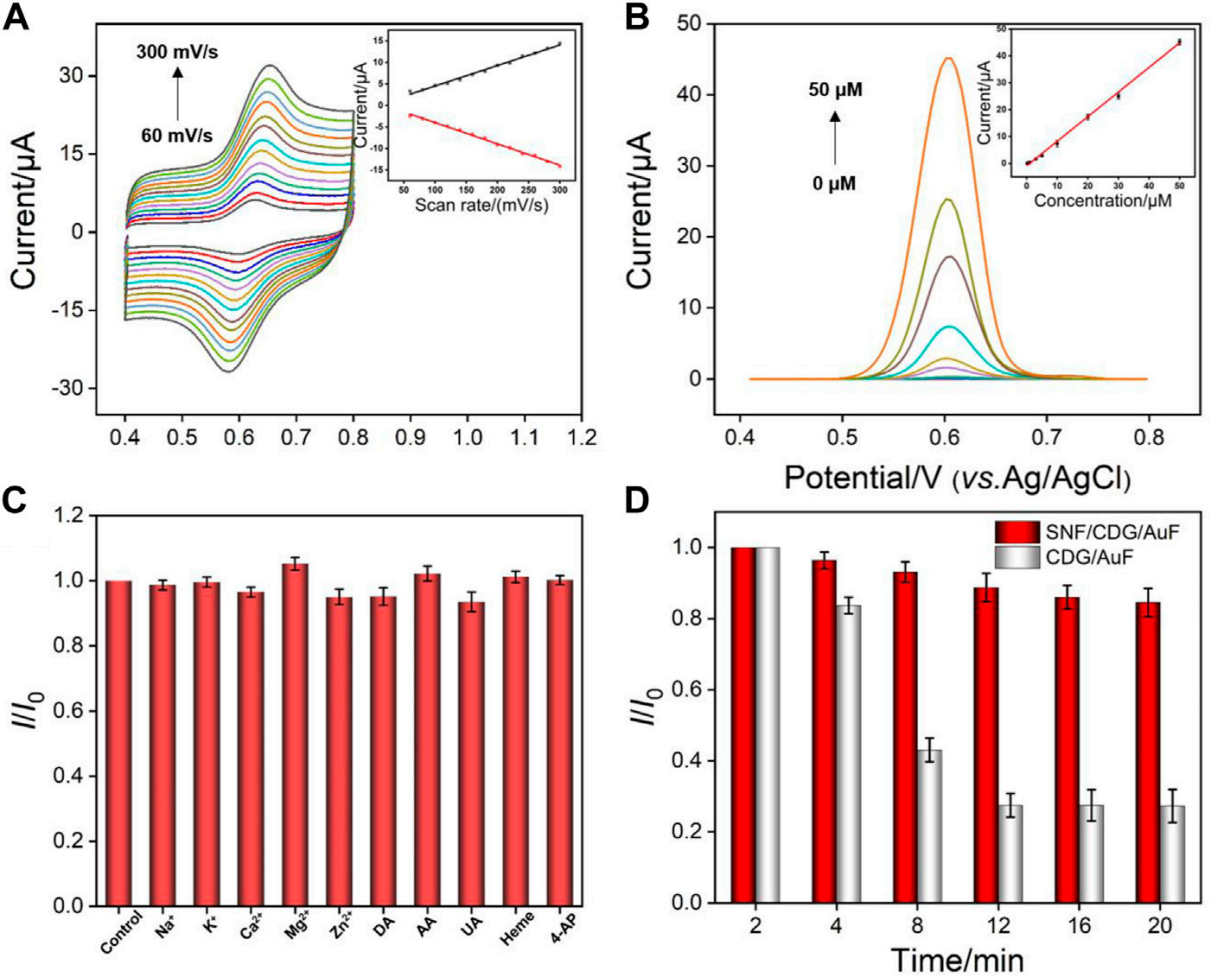
**(A)** CV curves on SNF/CDG/AuF for APAP (20 μM) at different scan rates after accumulation for 150 in PBS (0.1 M, pH 3.0). The inset shows the dependence of anodic and cathodic peak currents on the scan rate. **(B)** DPV curves on SNF/CDG/AuF with various concentrations of APAP. The inset shows the calibration curve. **(C)** Effect of interfering species on the anodic peak currents of APAP (5 μM) at the SNF/CDG/AuF electrode in PBS (0.1 M, pH 3.0). *I*_0_ and *I* depict the anodic peak currents measured in the absence and presence of interfering species (0.1 mM). Error bars denote the standard deviations of three measurements. **(D)** Anti-fouling performance of SNF/CDG/AuF and CDG/AuF in the paracetamol tablet matrix. *I*_0_ and *I* represent the anodic peak currents of APAP (20 μM) in continuous determination in different time.

Figure 6A shows the CV curves of APAP on SNF/CDG/AuF under different scan rates. The oxidation and reduction peak currents increase with increasing the scan rate, while the peak potential remains almost unchanged. The peak current is proportional to the scan rate, indicating an adsorption controlled electrochemical process. This further verifies the enrichment of APAP by CD and SNF.

### Sensitive Electrochemical Detection of APAP

Acetaminophen (APAP) is a commonly used drug for the treatment of colds. It can also relieve headache, joint pain, neuralgia, and other symptoms. As APAP is mainly synthesized by acetylation of p-aminophenol (4-AP), its product usually contains a small amount of unreacted 4-AP, which has the dual toxicity of both aniline and phenol. Specifically, 4-AP can cause methemoglobinemia and exhibits strong carcinogenicity through destroying DNA structure and causing gene mutation. Therefore, sensitive detection of APAP with high selectivity is of great significance.

Figure 6B shows DPV curves of SNF/CDG/AuF in presence of different concentrations of APAP. The oxidation peak current (*I*) is linearly correlated to the concentration of APAP (C) in the range from 0.2 to 50 μM (*I* = 0.904*C* - 0.943, *R* ^2^ = 0.997). A limit-of-detection (LOD) of 14 nM is obtained based on a signal-to-noise ratio of 3. Supplementary Table S1 (in Supplementary Materials) lists the performance comparison of different electrochemical sensors used to determine APAP. Attributable to synergistic enrichment from both CD and SNF nanochannels, LOD and the lowest concentration of the linear detection range from SNF/CDG/AuF sensor is lower than those obtained from nitrogen-rich porous carbon (P-NC) (Liang et al., 2019), Mo_2_C (Zhu et al., 2020), phosphorus-doped graphene (P-RGO) (Zhang et al., 2018), or SnO_2_-carbon carbon nanofiber (SnO_2_-CNF) (Hu et al., 2019), and Au nanoparticles@sulfourea-functionalized rGO (AuNPs@SFG) modified GCE (Singh et al., 2020).

We also investigated the selectivity of the developed sensor. As shown in Figure 6C, the peak current does not significantly change after adding various interferences. It is noteworthy that the response of APAP is almost unchanged even if the concentration of coexisting 4-AP is 20 times that of APAP, indicating high selectivity. The anti-interference performance of the sensor is also investigated. The detection in pharmaceutical tablets is used as a proof-of-concept to evaluate the detection in complex samples. Matrix in pharmaceutical tablets contains starch, sodium dodecyl sulfate, cellulose carboxylate, and other substances that might significantly interfere with detection. After APAP paracetamol tablets were ground into powder and dispersed in PBS buffer, the current signals were measured using both SNF/CDG/AuF and CDG/AuF (Figure 6D). As seen, the current on CDG/AuF decreases sharply in a short time. The signal at the 10th measurement is only 30% of the first measurement. On the contrary, SNF/CDG/AuF can maintain more than 80% of the original signals after 10 measurements, indicating high anti-fouling ability.

In combination of the anti-fouling ability of SNF, the developed SNF/CDG/AuF sensor is applied to detect APAP in commercially available paracetamol tablets. As shown in Supplementary Table S2 (in Supplementary Materials), the initial or artificial spiked concentrations of APAP in diluted extracting solution of APAP tablets obtained with the SNF/CDG/AuF are in good agreement with those obtained by high performance liquid chromatography (HPLC). This verifies the reliability of the SNF/CDG/AuF sensor in the detection of real samples.

## Conclusion

In summary, we prove that CDG nanocomposite can be used as a conductive nanoadhesive with enrichment effect to realize stable integration of SNF on AuF. In comparison with rGO/AuF without CD, the fabricated CDG/AuF sensor displays a higher oxidation peak, proving the enrichment effect of CD towards target analytes resulting from its host-guest complex. Combined with the enrichment of SNF nanochannels, the developed SNF/CDG/AuF sensor shows high sensitivity and can linearly detect APAP in the range from 0.2 to 50 μM with a limit-of-detection of 14 nM. In addition, high concentrations of AP do not affect the detection of APAP even when the concentration of 4-AP is 20 times of that of APAP, indicating good selectivity. Combined with the anti-fouling ability of SNF, the sensor can accurately and conveniently determine the content of APAP in commercially available paracetamol tablets. The work shows the feasibility of improving recognitive performance into SNF sensor by introducing a functional ligand on the electrode substrate. Through further seeking other compatible functional graphene nanocomposites as alternatives to CDG adhesive, the strategy might be extended to construct various SNF-graphene sensors for sensitive detection of a variety of electroactive molecules in complex samples.

## Data Availability

The original contributions presented in the study are included in the article/Supplementary Material, and further inquiries can be directed to the corresponding authors.
