# Supplementary material for: Silica Nanochannel Array Film Supported by ß-Cyclodextrin-Functionalized Graphene Modified Gold Film Electrode for Sensitive and Direct Electroanalysis of Acetaminophen
Source: Front Chem. 2022 Jan 13;9:812086. doi: 10.3389/fchem.2021.812086 (PMC8792962; doi:10.3389/fchem.2021.812086)
Supplement: Supplementary file 1 [file DataSheet1.docx]

Table S1 Comparison between detection performance of APAP using different electrochemical sensors.

| Electrode | Technique | Linear Range (μM) | LOD (nM) | Ref. |
| --- | --- | --- | --- | --- |
| P-NC/GCE | DPV | 3 - 110 | 500 | 32 |
| Mo_2_C/GCE | SWV | 0.5 - 600 | 29 | 33 |
| P-RGO/GCE^b^ | DPV | 1.5 - 120 | 360 | 34 |
| SnO_2_-CNF/GCE | DPV | 0.5 - 700 | 86 | 35 |
| AuNPs@SFG/GCE | DPV | 1.2 - 300 | 90 | 36 |
| SNF/CDG/GCE | DPV | 0.2 - 50 | 14 | This work |

P-NC, nitrogen-rich porous carbon; DPV, differential pulse voltammetry; SWV, square wave voltammetry; P-RGO, phosphorus-doped graphene; SnO_2_-CNF, doped SnO_2_ carbon nanofifiber; gold nanoparticles@sulfourea-functionalized reduced graphene oxide.

Table S2 Detection of APAP using HPLC or SNF/CDG/AuF sensor.

| Sample | Added  (μM) | Found by HPLC (μΜ) | Found by SNF/CDG/AuF (μΜ) |
| --- | --- | --- | --- |
| extracting solution of APAP tablets^a^ | - | 22.5 | 22.3 |
|  | 5.0 | 27.1 | 27.3 |
|  | 10.0 | 32.5 | 32.2 |
|  | 25.0 | 47.5 | 47.6 |

^a^ APAP tablets was extracted and detected as described in the experimental sections.
